# Prolactin in high‐metabolic risk pregnancies: Associations with maternal obesity and metabolic health

**DOI:** 10.1111/aogs.70248

**Published:** 2026-05-19

**Authors:** Kate Rassie, Simon Alesi, Adriana C. H. Neven, Taitum Mason, Eveline Jona, Stacey J. Ellery, Joanne Enticott, Aya Mousa, Anju E. Joham, David Simmons, Helena Teede, the TOBOGM Core Investigator Group

**Affiliations:** ^1^ Monash Centre for Health Research and Implementation (MCHRI), Faculty of Medicine, Nursing and Health Sciences Monash University Melbourne Victoria Australia; ^2^ Department of Diabetes Monash Health Melbourne Victoria Australia; ^3^ The Ritchie Centre Hudson Institute of Medical Research Melbourne Victoria Australia; ^4^ Department of Obstetrics and Gynaecology Monash University Melbourne Victoria Australia; ^5^ School of Medicine Western Sydney University Campbelltown New South Wales Australia

**Keywords:** breastfeeding, gestational diabetes, lactation, obesity, polycystic ovary syndrome, pregnancy in obesity, prolactin

## Abstract

**Introduction:**

Low prolactin levels during pregnancy have been linked to adverse maternal metabolic health. We examined pregnancy prolactin levels in relation to maternal metabolic characteristics within an ethnically diverse cohort at high metabolic risk.

**Material and Methods:**

In this observational cohort study, pregnant women (*n* = 130) with metabolic risk factors were characterized in early pregnancy, with sampling of serum prolactin and metabolic parameters. Fifty‐four women with gestational diabetes consented to serial profiling across pregnancy. Univariable and multivariable simple and mixed‐effects regression models examined relationships between prolactin and maternal variables.

**Results:**

Women with obesity (BMI ≥30 kg/m^2^) had lower early pregnancy prolactin levels than women without obesity (35.1 vs 44.3 μg/L, *p* = 0.03). Lower prolactin in early pregnancy was associated with higher parity and higher early pregnancy BMI, fasting insulin, insulin resistance, and diastolic blood pressure, but only relationships with parity and BMI persisted after adjustment for covariates. In the serially sampled cohort, lower prolactin levels across pregnancy were independently associated with higher pre‐pregnancy maternal BMI (adjusted *β* = −3.25, *p* = 0.04). Lower absolute prolactin increment from early to late pregnancy was independently associated with higher pre‐pregnancy and early pregnancy maternal BMI (*p* = 0.046 and *p* = 0.04, respectively).

**Conclusions:**

Maternal metabolic status, particularly higher BMI before and during early pregnancy, was associated with lower prolactin levels in early pregnancy and across gestation, and a smaller prolactin increase over pregnancy. Lower prolactin levels may reflect underlying metabolic health and may be relevant to the suboptimal lactation outcomes observed in women with obesity and metabolic disease.

## Graphical abstract

Higher maternal BMI before and during early pregnancy is associated with lower prolactin levels across gestation and a reduced prolactin rise. Prolactin in pregnancy may reflect maternal metabolic health and has potential relevance to lactation outcomes.

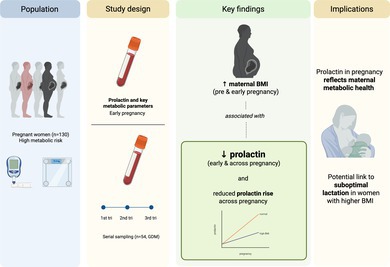

**Abbreviations**
BMI: body mass index
GDM: gestational diabetes mellitus
HOMA‐IR: homeostasis model assessment for insulin resistance
OGTT: oral glucose tolerance test
PCOS: polycystic ovary syndrome
TOBOGM: Treatment of Booking Gestational Diabetes Mellitus trial

**Key message**

In this high‐risk cohort, higher maternal BMI was associated with lower prolactin concentrations and a reduced gestational prolactin rise. Lower prolactin may reflect underlying maternal metabolic health in pregnancy, with potential relevance for subsequent lactation outcomes.

## 1 INTRODUCTION

Prolactin is a polypeptide hormone produced by lactotrophs in the anterior pituitary gland, and is the principal hormone of human lactation. It is present in circulation predominantly in its free, biologically active monomeric form. Beyond its reproductive functions, prolactin regulates various processes including glucose and lipid metabolism, and its role in human metabolic health is increasingly recognized.^1^ Experimental models (including during gestation) suggest bidirectional interactions between prolactin and insulin signaling: prolactin has been shown to directly impact β‐cell adaptation and insulin sensitivity, but metabolic status may also modulate prolactin secretion.^2^

Pathological hyperprolactinemia (such as in prolactinoma, or in patients treated with antipsychotic medications) is generally considered diabetogenic, and has been linked to obesity, impaired glucose tolerance and insulin resistance.^3^ However, lower prolactin levels within normal physiological ranges have also been associated with adverse metabolic profiles in large general population cohorts (e.g., insulin resistance^4^, ^5^, ^6^ and higher rates of incident^7^, ^8^ and prevalent^6^, ^9^, ^10^ type 2 diabetes mellitus and metabolic syndrome^11^, ^12^); findings which have been confirmed on systematic review.^13^ In non‐pregnant women with polycystic ovary syndrome (PCOS), a high metabolic risk population, lower prolactin levels within physiological ranges have consistently been associated with increased adiposity and an adverse metabolic phenotype.^14^, ^15^, ^16^

Evidence regarding the association between prolactin and metabolic health during pregnancy is more limited. Serum prolactin increases progressively across normal human pregnancy: peak values in late gestation are approximately 10‐fold higher than pre‐conception, reflecting progressive estrogen‐driven hyperplasia of pituitary lactotrophs.^17^, ^18^ Studies examining the relationship between prolactin and maternal body mass index (BMI) in pregnancy are few, and results are inconsistent. Some suggest that mothers with overweight/obesity have lower mid‐^19^ and late‐pregnancy^20^ prolactin levels, while others^21^, ^22^ report no association between maternal BMI and prolactin levels. Two recent Scandinavian studies have explored this further. Overgaard et al.^23^ showed that lower prolactin levels in both early and late pregnancy were associated with higher hemoglobin A1c (HbA1c), suggesting that low circulating prolactin should be considered a potential marker of metabolic risk during pregnancy. In that study, a lower relative prolactin increment from the first to the third trimester was also observed in pregnancies affected by PCOS (when compared with control pregnancies).^23^ Underdal et al.,^24^ in a secondary analysis of a randomized controlled trial of pregnant women with PCOS, found that a lower prolactin increment across pregnancy was associated with worse maternal metabolic health (higher BMI and systolic blood pressure, higher fasting glucose and insulin, and higher markers of insulin resistance). It should be noted that these studies were limited to White European populations, focusing on predominantly lean women^23^ or women with PCOS^24^; thus, the association between prolactin and metabolic health among ethnically and clinically diverse cohorts remains largely unknown. Indeed, our recent systematic review^25^ highlighted the ongoing paucity of high‐quality evidence examining the metabolic associations of prolactin in pregnancy, particularly early in gestation.

To address this gap, we aimed to examine pregnancy prolactin levels in relation to key maternal metabolic parameters within an ethnically diverse cohort of women with metabolic risk factors (cross‐sectionally in early pregnancy and longitudinally across pregnancy).

## 2 MATERIAL AND METHODS

### 2.1 Study design and participants

The current cohort represents a sub‐study of the Treatment of Booking Gestational Diabetes Mellitus (TOBOGM) trial.^26^ TOBOGM was an international multicenter randomized controlled trial investigating immediate treatment of early (<20 weeks) gestational diabetes mellitus (GDM) compared with standard care (treatment from 24 to 28 weeks). Women were recruited at the Monash Health/Monash University site in Melbourne, Australia, at between 4 weeks and 19 weeks + 6 days of gestation, based on at least one risk factor for hyperglycemia (previous GDM, BMI >30 kg/m^2^, age ≥40 years, first degree relative with diabetes, previous macrosomia, PCOS, or non‐European ancestry). Exclusion criteria included established pre‐gestational (Type 1 or Type 2) diabetes or other active medical disorders that local investigators considered a contraindication to participation. Psychiatric illness requiring treatment with antipsychotic medications was explicitly excluded as part of the latter category, which is relevant given the known potential for these agents to elevate prolactin levels.

This sub‐study was designed a priori and included in ethics submissions for the main trial. In total, 130 participants from the overall Monash cohort of 546 were selected using purposive sampling. This included all participants reporting a diagnosis of PCOS (*n* = 63) along with a random sample of those without the condition (*n* = 67). This sampling strategy was designed to enrich the cohort for PCOS subjects for broader biobank purposes and to allow for comparisons across clinically diverse groups; individual matching of participants was not performed. This sample constituted the baseline cohort (*n* = 130) for cross‐sectional analysis. A subset (*n* = 54) consented to additional blood sampling at 24–28 and 36 weeks of gestation and were also assessed longitudinally.

### 2.2 Clinical and anthropometric parameters

Clinical and anthropometric parameters for participants in the current analysis were measured at the first TOBOGM study visit (<20 weeks; mean gestation 13.9 ± 2.6 weeks, conducted between January 2018 and December 2019). Pre‐pregnancy weight, medical history and PCOS status were self‐reported on baseline study questionnaires, and a 2‐h 75 g oral glucose tolerance test (OGTT) was performed in all women as part of the main trial at <20 weeks (mean gestation 15.8 ± 2.5 weeks). Women in the current sub‐study had additional fasting bloodwork drawn at the same time for measurement of prolactin and additional metabolic parameters (*n* = 130).

For the longitudinal component, a subset of 54 of the 130 participants consented to further blood sampling in mid‐pregnancy (approx. 24–28‐week gestation) and late pregnancy (approx. 36‐week gestation) for prolactin and additional metabolic parameters. All 54 women included in the longitudinal analyses contributed blood samples at all three timepoints. Clinical and anthropometric measures were assessed in these women during the TOBOGM study visits at corresponding gestations. All 54 of these women had a diagnosis of GDM, either on the early OGTT at <20 weeks (*n* = 24) as part of the TOBOGM protocol, or at 24–28 weeks (*n* = 30) in keeping with routine care. GDM was defined according to the International Association of the Diabetes and Pregnancy Study Groups (IADPSG) diagnostic criteria^27^ and those with GDM received routine care including education, diet and lifestyle advice, written resources, and instructions on how to monitor capillary blood glucose levels four times daily. Thresholds for initiation and intensification of pharmacotherapy were as per the published TOBOGM trial,^28^ with insulin as first‐line treatment and metformin as a second‐line option in select cases.

### 2.3 Biochemical parameters and laboratory analysis

All venous blood samples were collected after an overnight fast. Results of the OGTTs were analyzed immediately at Monash Health Pathology per the TOBOGM protocol. Additional samples collected at the same time were frozen at −80°C until analysis, at which time they were thawed and analyzed batch‐wise at the Hudson Institute, Monash University.

Commercial enzyme‐linked immunosorbent assays (ELISA) were used per manufacturer's instructions to measure serum concentrations of prolactin and dehydroepiandrosterone (DHEA; Demeditec, Germany); androstenedione (IBL International, Germany); C‐peptide (Abcam, USA); and 5‐alpha‐dihydrotestosterone (DHT; Tecan, Germany). Human placental lactogen has previously been characterized in this cohort and reported separately.^29^ The Demeditec prolactin ELISA antibodies used in the assay kit detect a minimum human prolactin concentration of 0.35 ng/mL and have no cross‐reactivity with human chorionic gonadotropin, thyroid stimulating hormone, luteinising hormone, follicle stimulating hormone, or human growth hormone. Insulin and leptin concentrations were measured using a 20‐plex Luminex multi‐analyte kit (R&D Systems, USA) and testosterone using a 4‐plex Milliplex multi‐species hormone bead panel (Sigma Aldrich, Germany). All analyses were performed in duplicate, with the average of these measurements used to determine the final concentration. A consistent calibrator sample was included across all plates to account for batch effects and to determine inter‐ and intra‐assay coefficients of variation (CV).

The prolactin, DHEA, androstenedione, C‐peptide, and DHT assays each used six or seven standards, with intra‐assay CVs ranging from 3.35% to 11.76% and inter‐assay CVs ranging from 9.38% to 13.63%, within acceptable assay specifications. The insulin and leptin analyses, using a 20‐plex Luminex kit with seven standards, demonstrated intra‐ and inter‐assay CVs of 4.8% and 3.8% for insulin, and 4.6% and 2.0% for leptin, respectively. The 4‐plex Milliplex kit for testosterone, using seven standards, demonstrated intra‐ and inter‐assay CVs of 11.76% and 11.29%, respectively.

Plasma glucose was measured by Monash Health Pathology using a Hexokinase method on an AU5800 automated chemistry analyzer (Beckman Coulter, Brea, CA, USA). Area under the curve (AUC) for glucose was obtained using the trapezoidal rule.^30^ Homeostasis model assessment for insulin resistance (HOMA‐IR), a surrogate estimate of hepatic insulin resistance, was calculated as the product of fasting plasma insulin (FPI) and fasting plasma glucose (FPG), divided by the constant 22.5 (i.e., FPI [μU/mL] × FPG [mmol/L]/22.5).

### 2.4 Statistical analysis

For several metabolic analytes (prolactin, insulin, DHEA, DHT, and testosterone), visual inspection of the distribution of observations at each time point (using scatter plots and histograms) revealed up to two extreme upper outlying values. These were examined according to statistical rules for determining outlying values (three standard deviations [SD] above the mean for normally‐distributed variables,^31^ 1.5× the interquartile range [IQR] above the 75th centile for non‐normally distributed variables^31^, ^32^), with additional consideration given to pregnancy reference ranges for each time point. Values deemed extreme outliers were excluded from further analysis to avoid undue influence on the results.

Continuous variables were summarized as mean ± SD if normally distributed, or median with IQR for non‐normal distributions. Categorical outcomes were reported as counts and percentages. Differences in clinical and biochemical parameters according to maternal BMI category (≥30 vs <30 kg/m^2^) or PCOS status were assessed using independent samples *t*‐tests or Wilcoxon rank‐sum tests for normally or non‐normally distributed continuous variables, respectively, and chi‐squared tests were used for categorical variables.

In the cross‐sectional analysis (*n* = 130) at early pregnancy (<20 weeks gestation), univariable associations between maternal metabolic characteristics (e.g., fasting glucose, insulin, HOMA‐IR, etc.) and early pregnancy prolactin were assessed using simple linear regression, with early pregnancy prolactin as the dependent variable. For multivariable analysis, covariate selection was based on significance in the univariable analysis, the biological plausibility of an association with prolactin, and/or potential relevance based on the existing body of literature. To avoid collinearity, when variables were closely related (e.g., weight and BMI, or fasting insulin, and HOMA‐IR), only one was included in the model, prioritizing the variable deemed most clinically relevant.

For the longitudinal analysis (*n* = 54), mixed‐effects linear regression models were used to examine associations between prolactin levels and maternal clinical/metabolic parameters, with prolactin as the dependent variable. Univariable models assessed the relationship between prolactin, measured at three distinct time points (early, mid, and late pregnancy), and each maternal parameter individually. Time‐varying predictors (e.g., gestational age, insulin, C‐peptide, leptin, androgens) were modeled as fixed effects, while baseline characteristics (e.g., pre‐pregnancy BMI, age, parity, and baseline glycemic parameters) were included as time‐invariant covariates. Random effects were included to account for participant repeated measures. Parameters that showed significant univariable associations with prolactin were entered into a multivariable mixed‐effects model.

While mixed‐effects models provide insights into the trajectory of prolactin over pregnancy, the absolute change in prolactin between early and late pregnancy is conceptually important in understanding the potential impact of prolactin dynamics on lactation outcomes. A further simple linear regression examining the relationship between key maternal metabolic characteristics and absolute prolactin increment across pregnancy (dependent variable Δ prolactin, calculated as late pregnancy minus early pregnancy prolactin) was thus also performed.

A two‐tailed significance value of *p* < 0.05 was applied for all statistical tests. All analyses were performed using STATA software version 17.0 (StataCorp LLC, College Station, Texas, USA).

## 3 RESULTS

### 3.1 Sample characteristics

Participants in the cross‐sectional baseline cohort (*n* = 130; Table 1) had a mean age of 32.9 ± 4.4 years and a mean pre‐pregnancy BMI of 27.7 ± 8.5 kg/m^2^. The study cohort was ethnically diverse, comprising 43.8% South Asian, 23.1% White European, and 11.5% Southeast Asian, with remaining participants representing a variety of other ethnicities in smaller proportions. Of the 130 women, 54 (41.5%) were nulliparous prior to the index pregnancy. Twenty‐four reported a previous pregnancy affected by GDM (18.5% of the total cohort, 31.5% of the parous women), and almost half (48.5%) reported an existing diagnosis of PCOS.

**TABLE 1:** Baseline data at early pregnancy for the cohort overall and by first‐trimester BMI category.

Characteristics	All (*n* = 130)	BMI <30 (*n* = 89)	BMI ≥30 (*n* = 41)	*p*
Age at first trimester (years)	32.9 ± 4.4	32.7 ± 4.3	33.2 ± 4.5	0.60
Ethnicity				
White European	30 (23.1)			
Middle Eastern	4 (3.1)			
South Asian	57 (43.8)			
Southeast Asian	15 (11.5)			
East Asian	10 (7.7)			
Pacific Islander	2 (1.5)			
Other	12 (9.2)			
Parity prior to index pregnancy	0.8 ± 0.9	0.7 ± 0.7	1.0 ± 1.1	0.05
Nulliparous prior to index pregnancy (%)	54 (41.5)	38 (42.7)	16 (39.0)	0.12
Previous GDM (%)	24 (18.5)	19 (21.3)	5 (12.2)	0.39
Previous impaired glucose tolerance (%)	9 (7.3)	6 (7.1)	3 (7.5)	0.94
PCOS (%)	63 (48.5)	37 (41.6)	26 (63.4)	**0.02**
Family history of diabetes (%)	53 (41.1)	37 (42.0)	16 (39.0)	0.74
Previous macrosomia (%)	6 (4.6)	4 (4.5)	2 (4.9)	0.99
Gestation (weeks) at first clinical visit	13.9 ± 2.6	14.0 ± 2.5	13.6 ± 2.6	0.46
Gestation (weeks) at blood draw	15.8 ± 2.5	15.8 ± 2.5	15.6 ± 2.5	0.70
Weight pre‐pregnancy (kg)	73.5 ± 25.3	60.1 ± 9.0	102.6 ± 24.7	**<0.01**
BMI pre‐pregnancy (kg/m^2^)	27.7 ± 8.5	23.1 ± 3.1	37.9 ± 7.8	**<0.01**
Weight at first clinical visit (kg)	76.1 ± 25.1	62.9 ± 9.3	105.6 ± 24.1	**<0.01**
BMI at first clinical visit (kg/m^2^)	28.8 ± 8.4	24.1 ± 3.0	39.0 ± 7.4	**<0.01**
Systolic blood pressure at first clinical visit (mmHg)	111.1 ± 10.9	108.3 ± 10.0	118.0 ± 10.1	**<0.01**
Diastolic blood pressure at first clinical visit (mmHg)	67.2 ± 8.9	65.9 ± 7.9	70.6 ± 10.2	**0.01**
Fasting glucose (mmol/L)	4.6 ± 0.4	4.5 ± 0.4	4.7 ± 0.5	**0.01**
1 h glucose (mmol/L)	8.3 ± 2.1	8.2 ± 2.2	8.4 ± 1.9	0.74
2 h glucose (mmol/L)	7.1 ± 1.9	7.1 ± 2.0	7.0 ± 1.6	0.75
AUC glucose	14.1 ± 2.9	14.0 ± 3.1	14.2 ± 2.6	0.75
Fasting insulin (pmol/L)	38.3 ± 24.9	30.2 ± 17.6	55.6 ± 29.2	**<0.01**
HOMA‐IR	1.3 ± 0.9	1.0 ± 0.6	2.0 ± 1.1	**<0.01**
C‐Peptide (nmol/L)	0.42 (0.25, 0.60)	0.39 (0.24, 0.51)	0.51 (0.32, 0.83)	**0.01**
Prolactin (μg/L)	39.4 (25.1, 64.5)	44.3 (25.5, 71.9)	35.1 (21.3, 44.9)	**0.03**
Leptin (ng/mL)	9.9 (6.8, 15.0)	8.7 (6.2, 12.1)	17.3 (10.4, 22.8)	**<0.01**
Androstenedione (nmol/L)	9.0 (6.6, 13.0)	9.7 (6.8, 13.7)	8.7 (6.1, 10.8)	0.12
DHEA (nmol/L)	15.6 (11.5, 19.9)	16.4 (12.2, 21.1)	14.0 (11.1, 16.5)	**<0.01**
5‐Alpha dihydrotestosterone (nmol/L)	0.51 ± 0.32	0.45 (0.29, 0.60)	0.62 (0.43, 0.67)	**0.02**
Total testosterone (nmol/L)	4.5 ± 1.9	4.4 (3.2, 5.7)	4.2 (2.7, 5.4)	0.52

*Note*: Continuous variables are presented as mean ± SD if normally distributed, or median (IQR) if non‐normally distributed. Categorical variables are presented as *n* (%). Normally distributed variables were compared using two‐tailed *t*‐tests. Non‐normally distributed variables were compared using Wilcoxon Rank‐Sum tests. *p*‐value represents difference between BMI categories. Bold denotes statistical significance at two‐tailed *p* < 0.05.

Abbreviations: AUC, area under the curve; BMI, body mass index; DHEA, dehydroepiandrosterone; GDM, gestational diabetes mellitus; HOMA‐IR, homeostatic model assessment of insulin resistance; PCOS, polycystic ovary syndrome.

### 3.2 Early pregnancy prolactin levels overall and by BMI and PCOS status

Median early pregnancy prolactin in this cohort (sampled at mean 15.8 ± 2.5 weeks gestation) was 39.4 μg/L (IQR 25.1, 64.5). In the analysis grouped by first‐trimester maternal BMI (≥30 vs <30 kg/m^2^), women with obesity (*n* = 41) had significantly lower prolactin than those without obesity (*n* = 89), with median levels of 35.1 versus 44.3 μg/L, respectively (*p* = 0.03). Women with obesity also had higher systolic and diastolic blood pressures, higher levels of fasting glucose, insulin, leptin, C‐peptide, and 5α‐DHT, higher HOMA‐IR, and lower DHEA levels than those without obesity (Table 1). In analysis by PCOS status, early pregnancy prolactin was not significantly different between participants with PCOS (*n* = 62) and those without (*n* = 67) (median prolactin 34.2 vs 43.2 μg/L, respectively, *p* = 0.17).

### 3.3 Associations between early pregnancy metabolic parameters and early pregnancy prolactin

Univariable regression analysis showed a significant inverse relationship between early pregnancy prolactin and maternal BMI (both pre‐pregnancy and first trimester). Prolactin was also significantly inversely associated with parity prior to the index pregnancy, diastolic blood pressure, fasting insulin, and HOMA‐IR, and was positively associated with androstenedione (Table 2). There were no significant associations between prolactin and OGTT parameters: inverse relationships between prolactin and 1 h glucose and prolactin and AUC glucose did not reach statistical significance (*p* = 0.09 and 0.07, respectively). In the multivariable regression model, inverse relationships between prolactin and first‐trimester BMI and between prolactin and parity persisted following adjustment for covariates including maternal age, gestational age at blood draw, and fasting insulin (Table 2). These results did not change when HOMA‐IR was used in the model in place of fasting insulin.

**TABLE 2:** Univariable and multivariable associations between early pregnancy prolactin (dependent variable) and early pregnancy clinical and biochemical maternal characteristics.

(A) Univariable associations	Univariable prolactin (*n* = 130)	
	*β*	*p*
Age at first trimester (years)	−0.52	0.47
Parity prior to index pregnancy	−11.80	**<0.01**
Weight pre‐pregnancy (kg)	−0.34	**<0.01**
BMI pre‐pregnancy (kg/m^2^)	−1.05	**<0.01**
Weight at first clinical visit (kg)	−0.36	**<0.01**
BMI at first clinical visit (kg/m^2^)	−1.14	**<0.01**
Systolic blood pressure at first clinical visit (mmHg)	−0.53	0.06
Diastolic blood pressure at first clinical visit (mmHg)	−0.64	**0.048**
Gestation (weeks) at blood draw	5.79	**<0.01**
Fasting glucose (mmol/L)	−6.13	0.21
1 h glucose (mmol/L)	−2.28	0.09
2 h glucose (mmol/L)	−2.17	0.13
AUC glucose	−1.66	0.07
Fasting insulin (pmol/L)	−0.19	**0.04**
HOMA‐IR	−5.25	**0.03**
C‐Peptide (nmol/L)	−7.60	0.23
Leptin (ng/mL)	−0.61	0.09
Androstenedione (nmol/L)	1.23	**0.01**
DHEA (nmol/L)	0.94	0.07
5‐Alpha dihydrotestosterone (nmol/L)	−8.75	0.31
Total testosterone (nmol/L)	2.89	0.05

(B) Multivariable model	Multivariable prolactin (*n* = 130)	
	*β*	*p*
Age at first trimester (years)	0.68	0.34
Parity prior to index pregnancy	−11.54	**<0.01**
BMI at first clinical visit (kg/m^2^)	−0.97	**0.01**
Gestation (weeks) at blood draw	5.46	**<0.01**
Fasting insulin (pmol/L)	0.05	0.70

*Note*: Data are presented as *β*‐coefficients with corresponding *p*‐values, representing the relationships with prolactin (A) before adjustment, using univariable simple linear regression, and (B) after adjustment for the other variables in the model, using multivariable linear regression. Bold denotes statistical significance at two‐tailed *p* < 0.05.

Abbreviations: AUC, area under the curve; BMI, body mass index; DHEA, dehydroepiandrosterone; HOMA‐IR, homeostatic model assessment of insulin resistance.

### 3.4 Associations between early pregnancy metabolic parameters and prolactin across pregnancy

The serially sampled cohort comprised 54 women with repeated measures at early, mid, and late pregnancy, with blood draws at mean gestational ages of 16.0 ± 2.5, 25.8 ± 1.4, and 35.9 ± 1.4 weeks, respectively (Table 3). Characteristics of this subset of participants were similar to the overall cohort in relation to maternal pre‐pregnancy BMI (27.0 vs 27.7 kg/m^2^, *p* = 0.49), early pregnancy BMI (28.1 vs 28.8 kg/m^2^, *p* = 0.51), and parity (35.2% vs 41.5% nulliparous prior to index pregnancy, *p* = 0.34). Maternal age was slightly older in the serially sampled subset than in the overall cohort (34.3 vs 32.9 years, *p* = 0.03), although this difference was unlikely to be clinically significant. Of the 54 participants, *n* = 23 had GDM managed with dietary modification alone, *n* = 30 had GDM requiring pharmacotherapy (insulin and/or metformin), and *n* = 1 had GDM with unknown treatment status.

**TABLE 3:** Characteristics across pregnancy for the serially sampled cohort, *n* = 54.

Characteristics	Early pregnancy	Mid pregnancy	Late pregnancy
	<20 weeks	22–29 weeks	32–38 weeks
	(*n* = 54)	(*n* = 54)	(*n* = 54)
Age at first trimester (years)	34.3 ± 4.7	—	—
Parity prior to index pregnancy	0.9 ± 0.9	—	—
Nulliparous prior to index pregnancy (%)	19 (35.2)	—	—
Previous GDM (%)	11 (20.4)	—	—
Previous impaired glucose tolerance (%)	4 (7.7)	—	—
PCOS (%)	13 (24.1)	—	—
Family history of diabetes (%)	23 (43.4)	—	—
Previous macrosomia (%)	1 (1.9)	—	—
Weight pre‐pregnancy (kg)	71.4 ± 22.8	—	—
BMI pre‐pregnancy (kg/m^2^)	27.0 ± 7.6	—	—
Gestation (weeks) at clinical visit	14.2 ± 2.6	25.9 ± 1.6	35.6 ± 1.2
Weight at clinical visit (kg)	74.2 ± 22.5	75.4 ± 15.6	80.5 ± 20.4
BMI at clinical visit (kg/m^2^)	28.1 ± 7.4	28.7 ± 5.3	30.7 ± 6.5
Systolic blood pressure at clinical visit (mmHg)	111.2 ± 9.9	108.4 ± 9.6	113.6 ± 9.8
Diastolic blood pressure at clinical visit (mmHg)	68.6 ± 9.5	67.0 ± 8.1	70.5 ± 8.9
Gestation (weeks) at OGTT	16.0 ± 2.5	—	—
Fasting glucose (mmol/L)	4.8 ± 0.4	—	—
1 h glucose (mmol/L)	9.3 ± 2.1	—	—
2 h glucose (mmol/L)	7.9 ± 1.7	—	—
AUC glucose	15.6 ± 2.7	—	—
HOMA‐IR	1.2 ± 0.8	—	—
Gestation (weeks) at blood draw	16.0 ± 2.5	25.8 ± 1.4	35.9 ± 1.4
Prolactin (ug/L)	42.9 (26.8, 63.1)	84.2 (55.7, 131.0)	100.2 (76.3, 163.1)
Fasting insulin (pmol/L)	30.7 (19.1, 44.4)	37.2 (24.8, 62.4)	31.1 (21.3, 51.5)
C‐peptide (nmol/L)	0.41 (0.27, 0.58)	0.49 (0.34, 0.69)	0.49 (0.37, 0.67)
Leptin (ng/mL)	10.1 (6.5, 14.8)	11.2 (7.0, 15.7)	10.1 (5.5, 15.2)
Androstenedione (nmol/L)	7.5 (6.1, 12.2)	8.4 (6.6, 10.8)	11.0 (8.5, 15.0)
DHEA (nmol/L)	16.1 (11.5, 23.5)	15.2 (11.7, 20.3)	19.8 (15.7, 26.6)
5‐Alpha dihydrotestosterone (nmol/L)	0.47 (0.24, 0.64)	0.52 (0.30, 0.67)	0.67 (0.49, 0.82)
Total testosterone (nmol/L)	4.3 ± 1.6	4.7 ± 1.8	6.9 ± 3.4

*Note*: Continuous variables are presented as mean ± SD if normally distributed, or median (IQR) if non‐normally distributed. Categorical variables are presented as *n* (%).

Abbreviations: AUC, area under the curve; BMI, body mass index; DHEA, dehydroepiandrosterone; GDM, gestational diabetes mellitus; HOMA‐IR, homeostatic model of insulin resistance; OGTT, oral glucose tolerance test; PCOS, polycystic ovary syndrome.

In univariable mixed‐effects linear regression analysis (Table 4), there was a clear positive relationship between maternal prolactin levels and gestational age (in weeks) at the time of blood sampling, consistent with the known upward trajectory of prolactin levels across gestation. Lower prolactin across pregnancy was associated with higher maternal BMI prior to pregnancy and in early pregnancy (*β* = −3.32 and *p* = 0.03 for pre‐pregnancy BMI; *β* = −3.53 and *p* = 0.02 for first‐trimester BMI). There was an inverse relationship between prolactin across pregnancy and parity prior to the index pregnancy, and a positive relationship between prolactin levels across pregnancy with each of the four maternal androgens measured (androstenedione, DHEA, 5‐alpha dihydrotestosterone, and total testosterone), expected given that maternal serum levels of these analytes also increase across human gestation.^33^ In the multivariable model, however, only the positive association between prolactin and gestational age at blood draw (*β* = 5.48, *p* < 0.001), and the inverse association between prolactin and maternal pre‐pregnancy BMI (*β* = −3.25, *p* = 0.04) remained significant following adjustment for other covariates. Late pregnancy prolactin was not significantly different between participants with diet‐controlled GDM (median prolactin 95.2 μg/L, *n* = 22) and those with GDM requiring pharmacotherapy (median prolactin 104.6 μg/L, *n* = 29; *p* = 0.77).

**TABLE 4:** Univariable and multivariable mixed‐effects linear regression model examining associations between prolactin across pregnancy (dependent variable) and clinical/biochemical maternal characteristics, *n* = 54 (serially sampled cohort).

(A) Univariable model	*β*	*p*‐value
Age at first trimester (years)	−0.56	0.83
Parity prior to index pregnancy	−26.6	**0.04**
Weight pre‐pregnancy (kg)	−1.14	**0.02**
BMI pre‐pregnancy (kg/m^2^)	−3.32	**0.03**
Weight at first clinical visit (kg)	−1.17	**0.02**
BMI at first clinical visit (kg/m^2^)	−3.53	**0.02**
Systolic blood pressure at corresponding clinical visit (mmHg)	−0.53	0.55
Diastolic blood pressure at corresponding clinical visit (mmHg)	0.50	0.60
Fasting glucose (mmol/L)	−16.9	0.55
1 h glucose (mmol/L)	−9.14	0.11
2 h glucose (mmol/L)	−3.89	0.58
AUC glucose	−6.28	0.15
HOMA‐IR	−2.23	0.69
Gestation (weeks) at blood draw	6.53	**<0.001**
Fasting insulin (pmol/L)	−0.20	0.57
C‐peptide (nmol/L)	9.96	0.72
Leptin (ug/L)	−0.17	0.92
Androstenedione (nmol/L)	5.59	**0.001**
DHEA (nmol/L)	2.73	**0.01**
5‐Alpha dihydrotestosterone (nmol/L)	73.4	**0.005**
Total testosterone (nmol/L)	17.08	**<0.001**

(B) Multivariable model	*β*	*p*‐value
Parity prior to index pregnancy	−19.4	0.15
BMI pre‐pregnancy (kg/m^2^)	−3.25	**0.04**
Gestation (weeks) at blood draw	5.48	**<0.001**
Androstenedione (nmol/L)	0.92	0.63
DHEA (nmol/L)	−0.15	0.91
5‐Alpha dihydrotestosterone (nmol/L)	39.0	0.14
Total testosterone (nmol/L)	5.57	0.24

*Note*: Data are presented as *β*‐coefficients with corresponding *p*‐values. In (A), results represent the relationship between prolactin, as the dependent variable, and each predictor individually. (B) Results of a multivariable model with prolactin as the dependent variable. Time‐varying predictors (gestational age, insulin, C‐peptide, leptin, androgens) were measured at three distinct time points: early (visit 1), mid (visit 2), and late (visit 3) pregnancy, as was prolactin. Baseline characteristics (e.g., weight/BMI, age, parity, and oral glucose tolerance test parameters) were measured in early pregnancy only. Models accounted for within‐subject correlation by including random intercepts for each participant. Estimates represent fixed effects, with statistical significance defined as two‐tailed *p* < 0.05 (denoted in bold).

Abbreviations: AUC, area under the curve; BMI, body mass index; DHEA, dehydroepiandrosterone; HOMA‐IR, homeostatic model of insulin resistance.

Median prolactin increment across pregnancy (Δ prolactin, calculated as late pregnancy minus early pregnancy prolactin) was 64.2 μg/L (*n* = 52, following removal of outliers). For between‐group comparisons by BMI category, the magnitude of prolactin increment was significantly lower in women with obesity prior to pregnancy than those without (median Δ prolactin 37.2 μg/L, *n* = 11, vs 74.4 μg/L, *n* = 41, respectively; *p* = 0.02). This difference did not reach statistical significance when maternal first‐trimester BMI (as opposed to pre‐pregnancy BMI) was used to categorize the groups (median 38.2 μg/L, *n* = 14 vs 74.0 μg/L, *n* = 38, respectively; *p* = 0.07). In analysis by PCOS status, prolactin increment across pregnancy did not differ significantly between women with PCOS (median 74.4 μg/L, *n* = 13) and those without (median 60.8 μg/L, *n* = 39; *p* = 0.63).

**FIGURE 1:**
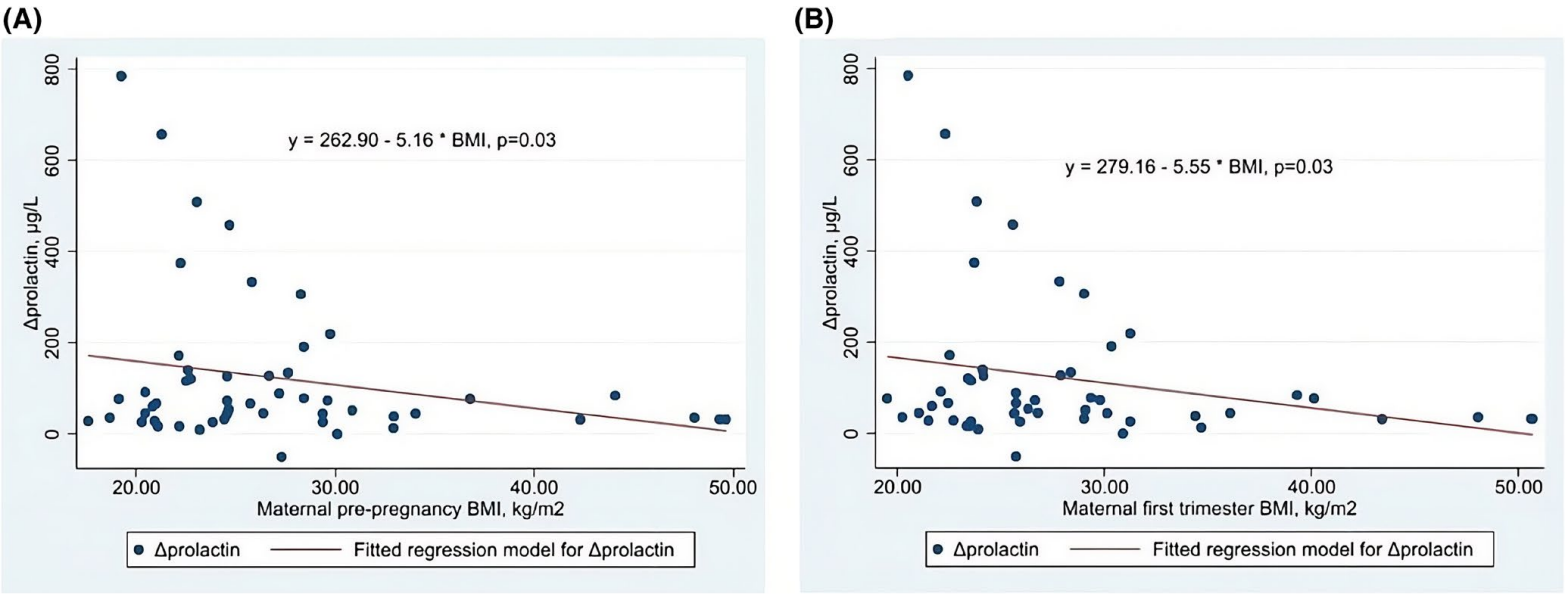
Scatter plots depicting linear regression models for change in prolactin (Δ prolactin) across pregnancy in relation to (A) maternal pre‐pregnancy body mass index and (B) maternal first‐trimester body mass index (serially sampled cohort). BMI, body mass index.

Univariable regression, with Δ prolactin as the dependent variable, showed that lower prolactin increment across pregnancy was significantly correlated with both higher pre‐pregnancy and first‐trimester BMI (Table 5; Figure 1). These relationships remained significant after correction for maternal age and parity (*β* = −4.73 and *p* = 0.046 for pre‐pregnancy BMI; *β* = −5.02 and *p* = 0.04 for first‐trimester BMI). Lower prolactin increment across pregnancy was also associated with higher early pregnancy systolic blood pressure on univariable analysis (*β* = −6.50, *p* = 0.03). No other early pregnancy variables were significantly associated with prolactin increment.

**TABLE 5:** Univariable associations between prolactin increment across pregnancy (Δ prolactin, dependent variable) and baseline clinical/biochemical maternal characteristics (serially sampled cohort, *n* = 54).

Univariable Δ prolactin	*β*	*p*
Age at first trimester (years)	−2.06	0.52
Parity prior to index pregnancy	−28.16	0.12
Weight pre‐pregnancy (kg)	−1.84	**0.04**
BMI pre‐pregnancy (kg/m^2^)	−5.16	**0.03**
Weight at first clinical visit (kg)	−1.91	**0.04**
BMI at first clinical visit (kg/m^2^)	−5.55	**0.03**
Systolic blood pressure at first clinical visit (mmHg)	−6.50	**0.03**
Diastolic blood pressure at first clinical visit (mmHg)	−3.83	0.19
Fasting glucose (mmol/L)	3.08	0.95
1 h glucose (mmol/L)	−13.15	0.25
2 h glucose (mmol/L)	−4.13	0.72
AUC glucose	−8.34	0.34
HOMA‐IR	−16.63	0.49
Fasting insulin (pmol/L)	−0.56	0.56
C‐peptide (nmol/L)	95.5	0.24
Leptin (ug/L)	−0.14	0.95
Androstenedione (nmol/L)	−0.14	0.98
DHEA (nmol/L)	4.84	0.15
5‐Alpha dihydrotestosterone (nmol/L)	−45.15	0.28
Total testosterone (nmol/L)	0.39	0.98

*Note*: Data from univariable analysis using simple linear regression are presented as *β*‐coefficients with corresponding *p*‐values. Coefficients describe the relationship between change in prolactin over pregnancy (Δ prolactin, dependent variable) and each maternal clinical/biochemical measure at early pregnancy. Bold denotes statistical significance at two‐tailed *p* < 0.05.

Abbreviations: AUC, area under the curve; BMI, body mass index; DHEA, dehydroepiandrosterone; HOMA‐IR, homeostatic model of insulin resistance.

## 4 DISCUSSION

Our findings show that low prolactin levels during early pregnancy are associated with markers of increased maternal metabolic risk, including higher BMI, elevated diastolic blood pressure, and increased fasting insulin levels and insulin resistance. The inverse relationship between prolactin and BMI persisted across gestation, independent of other covariates, and higher maternal BMI at either pre‐pregnancy or early pregnancy was independently associated with reduced prolactin increment from early to late pregnancy.

Our recent systematic reviews^25^, ^34^ highlighted significant knowledge gaps regarding the associations between prolactin and maternal metabolic health in pregnancy, including a lack of published data on early pregnancy prolactin in relation to metabolic health (studies to date have focused on prolactin in mid‐late pregnancy, particularly in relation to maternal GDM status).^25^ The relationship between maternal BMI and prolactin has been unclear, although small studies in Korean^19^ and Chinese^20^ cohorts suggest lower mid‐ and late‐pregnancy prolactin levels (respectively) in women with overweight/obesity. Our data extend these observations by showing, in a well‐characterized and more ethnically diverse cohort, that higher maternal BMI was associated with lower prolactin levels in early pregnancy and across gestation.

Our findings are also consistent with the two Scandinavian studies by Underdal et al.^24^ and Overgaard et al.,^23^ mentioned earlier, both of which suggested that low serum prolactin in pregnancy may also be a broader marker of maternal metabolic risk. The study by Underdal et al.^24^ explored a cohort where all participants had PCOS, finding that lesser prolactin increase across pregnancy was associated with increased BMI and systolic blood pressure at week 32, and lesser breast size increment across pregnancy. In their cohort, a prolactin increase below the median was associated with inferior metabolic health (higher fasting glucose, fasting insulin, and HOMA‐IR). Similarly, Overgaard et al.^23^ reported an inverse relationship between prolactin and HbA1c across pregnancy in a large community cohort and showed a lower relative increase in prolactin across pregnancy in PCOS compared with controls. However, this study was limited to a predominantly lean, ethnically homogenous cohort, where BMI was reported only as a covariate in the analyses. Here, we add to current evidence by demonstrating similar associations for the first time in an ethnically diverse, high‐metabolic risk cohort (including participants with and without PCOS). Further, our focus on early pregnancy associations suggests that maternal metabolic status prior to pregnancy (particularly BMI) is independently associated with both early pregnancy prolactin and subsequent prolactin increase.

The metabolic associations of prolactin in pregnancy are highly relevant from a lactation perspective. Maternal metabolic dysregulation adversely impacts breastfeeding outcomes: observational evidence from our group^35^ and others^36^ demonstrates that women with pre‐gravid obesity breastfeed at reduced rates and durations compared with their normal‐weight peers, and recent work directly implicates insulin resistance in the pathophysiology of low milk supply.^37^, ^38^, ^39^ Women with obesity and/or diabetes are at increased risk of lactogenesis delay^40^, ^41^ and, in the post‐partum period, there is evidence for impaired prolactin responses to suckling in mothers with obesity.^42^ Data from the wider TOBOGM cohort (*n* = 3323) also demonstrated a reduced likelihood of breastfeeding as first feeding method among mothers with late recognition and treatment of GDM compared to mothers without GDM (adjusted odds ratio [aOR] = 0.62, *p* = 0.001). The effect appeared to be alleviated by early diagnosis and treatment of GDM, likely reflecting the benefits of early, sustained euglycemia for mammary gland function.^43^

The specific contribution of abnormal prolactin dynamics during pregnancy to the suboptimal breastfeeding outcomes observed in women with metabolic disease remains unclear. However, prolactin is critical to breast development from the earliest stages of pregnancy (acting directly on mammary epithelial cells to initiate alveologenesis, then—as pregnancy progresses—facilitating alveolar secretory differentiation). Across pregnancy, the rise in maternal serum prolactin correlates with mammary synthetic activity.^44^ As such, it is plausible that lower early gestational prolactin and a reduced prolactin increment across pregnancy may contribute to the suboptimal lactation performance observed in women with obesity and/or metabolic disease. Prolactin increment across pregnancy has been shown to correlate directly with breast size increase,^24^ and, in the lactation literature, breast hypoplasia/insufficient glandular tissue is commonly linked to both PCOS and obesity in association with suboptimal lactation performance.^45^, ^46^, ^47^ Our study supports this hypothesis by showing that early pregnancy prolactin is inversely associated with maternal BMI, fasting insulin, and HOMA‐IR; and that prolactin increment across pregnancy is inversely associated with maternal BMI.

Taken together, current observational evidence suggests that the relationship between lactation and long‐term maternal metabolic health is likely complex. The “reset hypothesis” proposes that breastfeeding itself induces favorable metabolic adaptations—including improvements in glucose and lipid metabolism—that persist beyond the period of active lactation. This hypothesis is supported by extensive observational evidence suggesting that breastfeeding, particularly for an extended duration, is associated with reduced maternal risk of developing pre‐diabetes and type 2 diabetes,^48^, ^49^ as well as reduced cardiovascular risk.^50^ Our findings lend support to an alternative “preset” hypothesis, which suggests that such observations may be explained, at least in part, by reverse causality: that is, that poor maternal metabolic health prior to and during pregnancy leads to impaired breastfeeding ability and reduced breastfeeding durations (mediated, as our study would suggest, by reduced gestational prolactin levels and suboptimal prolactin dynamics, in combination with insulin resistance). Notably, recent postpartum studies have also reported associations between lower prolactin concentrations and increased risk of type 2 diabetes among women with prior GDM, supporting the possibility that prolactin biology may intersect with metabolic risk in the early postpartum period.^51^ As such, underlying maternal metabolic health may influence both lactation performance and longer‐term metabolic trajectories.^24^, ^52^ The “reset” and “preset” hypotheses are not necessarily mutually exclusive: it is plausible that poorer pre‐pregnancy metabolic health may impair the initiation or establishment of lactation, while sustained lactation may independently confer metabolic benefit during and after the breastfeeding period. Further research is required to disentangle these pathways.

Our study also demonstrates an inverse relationship between early pregnancy prolactin and parity prior to the index pregnancy, which remained significant following adjustment for key covariates in the multivariate model. Univariate analysis in the serially‐sampled cohort was also suggestive of an ongoing association between parity and prolactin levels across pregnancy. This finding was entirely consistent with existing literature: a similar relationship between higher parity and lower circulating prolactin levels has been consistently reported in non‐pregnant premenopausal women,^7^, ^53^, ^54^, ^55^, ^56^ and has also been described in lactating women in the early post‐partum period.^57^ Parity was not specifically considered in relation to prolactin in the recent studies by Underdal^24^ or Overgaard,^23^ and was not adjusted for as a covariate in regression analyses in either of these studies. Our findings indicate that parity should be regarded as an important potential confounder in future research on gestational prolactin levels.

Strengths of this study include our well‐profiled and ethnically diverse cohort of high metabolic risk pregnancies; reflective of a contemporary, multicultural urban population and enriched for established GDM risk factors. Serial measurement of analytes across pregnancy in a subset of women allowed us to assess gestational trajectories. Our multivariable models were adjusted for exact gestational age at the time of sampling, which is critical when analyzing a parameter with a steep upward trajectory across gestation. Limitations include the modest sample size overall (particularly for the serially sampled cohort), the lack of repeated OGTT data beyond early pregnancy to pair with serial prolactin measures, the self‐reported nature of PCOS status, and the restriction of the serially sampled cohort to women affected by GDM (with heterogeneity in pharmacotherapy that could not be meaningfully accounted for in our analyses). As longitudinal sampling was limited to a subset of participants with ongoing trial contact, findings from the serial analyses may not be fully generalizable to the broader cohort and should be interpreted with appropriate caution. Accordingly, results should be interpreted as exploratory, with effect sizes and biological plausibility considered alongside statistical significance. Although prolactin was first measured at a mean of 15.8 weeks' gestation, earlier rises may have occurred prior to this timepoint and were not captured. Finally, absence of specific data on breast size increase or lactation outcomes means the implications of our findings for lactation remain theoretical.

## 5 CONCLUSION

In conclusion, higher maternal BMI in early and/or pre‐pregnancy was independently associated with lower prolactin levels in early pregnancy, lower prolactin levels across pregnancy (assessed using a mixed‐effects model), and a smaller absolute rise in prolactin from early to late pregnancy. Lower gestational prolactin levels may be a marker of adverse maternal metabolic health, and further research is required to elucidate the implications of these findings for lactation in women with obesity and metabolic disease.

## AUTHOR CONTRIBUTIONS

KR conceptualized the analysis, with oversight from AM, AEJ, and HT. HT led the overall TOBOGM study at the Monash site, with trial co‐ordination by EJ and clinical support from ACHN. Laboratory analysis for the current study was led by SA, with support from TM and oversight from SJE. Statistical analyses were performed by KR, with expert biostatistical oversight from JE. KR drafted the manuscript, which was reviewed and revised by JE, DS, AM, AEJ, and HT, and approved by all other authors. All authors have contributed to this manuscript in line with the ICMJE criteria for authorship and have read and approved the manuscript for publication. This manuscript represents original work, and its contents have not been submitted or published elsewhere.

## FUNDING INFORMATION

This research did not receive any specific grant from any funding agency in the public, commercial or not‐for‐profit sector. KR is supported by a scholarship from the Australian National Health and Medical Research Council (NHMRC). SA and ACHN are supported by Monash University scholarships. SJE, AM, AEJ, and HT are supported by fellowships from the NHMRC.

## CONFLICT OF INTEREST STATEMENT

All authors report no conflicts of interest.

## ETHICS STATEMENT

The TOBOGM study was registered (ACTRN12616000924459) and had ethics approval from the South Western Sydney Local Health District Human Research Ethics Committee (reference 15/LPOOL/551, approval 9/11/2015), and locally from the Monash Health Research Ethics Committee (reference RES‐17‐0000‐406X, approval 6/4/2016). Full study protocol details have been published elsewhere.^58^ All participants provided informed written consent for participation.

## Supporting information


**Supporting Information S1.** TOBOGM Core Investigator Group.

## Data Availability

A data sharing statement relevant to the broader TOBOGM study (from which all clinical, anthropometric, and OGTT data were sourced) is available in relation to that published study.^58^ Biochemical data (prolactin levels and metabolic analytes) are not currently publicly available but can be obtained from the corresponding author upon reasonable request. Data access will be granted to qualified researchers for purposes of replicating or extending the study, pending institutional approval where required.
